# A Planar-Gate Graphene Field-Effect Transistor Integrated Portable Platform for Rapid Detection of Colon Cancer-Derived Exosomes

**DOI:** 10.3390/bios15040207

**Published:** 2025-03-24

**Authors:** Zaiyu Zhang, Luyang Zhang, Yuting Huang, Ziran Wang, Zhongjing Ren

**Affiliations:** 1 Key Laboratory of High-Efficiency and Clean Mechanical Manufacture, Ministry of Education, Department of Mechanical Engineering, Shandong University, Jinan 250100, China; 202334481@mail.sdu.edu.cn (Z.Z.);; 2 Department of Clinical Laboratory, Qilu Hospital of Shandong University, Jinan 250012, China; 3 Shandong Engineering Research Center of Biomarker and Artificial Intelligence Application, Jinan 250012, China

**Keywords:** GFET biosensor, polydopamine, exosome detection, colon cancer diagnosis

## Abstract

Early diagnosis of diseases would significantly increase the survival rate of cancer patients. However, current screening methods are complex and costly, making them unsuitable for rapid health diagnosis in daily life. Here, we develop a portable platform based on a planar-gate graphene field-effect transistor functionalized with polydopamine self-assembled film (PDA-GFET), capable of identifying colon cancer through the detection of EpCAM protein, which is expressed on colon cancer-derived exosomes, in clinical samples within 10 min. The PDA self-assembled film on the graphene and gate surface enhances the biosensor’s functionalization area while suppressing non-specific adsorption, thereby achieving detection limits as low as 112 particles/mL. In addition, the PDA-GFET-based detection platform was used to identify EpCAM protein in real clinical samples from healthy individuals and colon cancer patients within 10 min, and the two showed significant differences (*p* < 0.001). Results indicate that the proposed PDA-GFET-based detection platform is expected to be a potential tool for the early diagnosis of colon cancer.

## 1. Introduction

Colon cancer is one of the most commonly diagnosed cancers globally, ranking as the third highest in terms of incidence, with a higher frequency of occurrence than rectal cancer [1,2,3]. Early-stage colon cancer often has no obvious symptoms (e.g., intestinal bleeding, etc.), resulting in it being detected mostly at a late stage, which seriously affects cure rate [4,5,6]. Although techniques such as colonoscopy [7], immunoassay [8], and fluorescence [9] are widely used in clinical settings for colorectal cancer detection, their bulky instruments and complicated processes make them unsuitable for health diagnosis in daily life. Therefore, it is crucial to develop a novel measurement method to achieve early diagnosis of colon cancer.

Exosomes are widely present in bodily fluids such as blood, urine, and saliva [10,11,12], which carry a variety of biomarkers (e.g., proteins, RNAs, miRNAs) in their outer membrane and contents [13,14]. In colon cancer, exosomes are derived from colon cancer initiating cells and play an important role in the signaling of the tumor microenvironment and the regulation of cancer cell function [15,16]. Specific molecular markers carried by exosomes (e.g., EpCAM, CD44, CD133, ALDH1) are considered to have potential as early diagnostic markers for colon cancer [17,18,19]. Studies have shown that EpCAM is specifically enriched on exosomes derived from cancer cells, with its high expression correlating with the invasiveness and malignancy of colorectal tumors [20,21]. Therefore, EpCAM exhibits both high specificity and sensitivity in colorectal cancer, and it is expected to provide important clues for the early diagnosis of colon cancer.

Graphene, a two-dimensional material, has high carrier mobility and sensitivity to surface charge [22,23,24,25]. The aptamer is a class of nucleic acid molecules obtained through in vitro screening (SELEX) that can exhibit high selectivity for specific target molecules [26,27], such as exosome surface markers [28,29]. In recent years, graphene-based field-effect transistors (GFETs) have attracted enormous interest [30]; the combination of GFETs and aptamers (aptamer-GFETs) has gained much attention in the field of biosensing [23,31,32,33]. Due to the specificity of the aptamer to the target molecule, aptamer-GFET shows great potential for rapid and sensitive detection of biomolecules such as cytokines, large proteins, and viruses. However, aptamer-GFET still faces challenges such as poor resistance to nonspecific adsorption [34,35], insufficient sensing performance in real clinical samples [36,37], and complicated electrical testing equipment [38,39,40], which make it difficult to achieve practical application in convenient and accurate detection of disease biomarkers in daily life, requiring significant improvement of their detection ability for practical applications.

**Figure 1:**
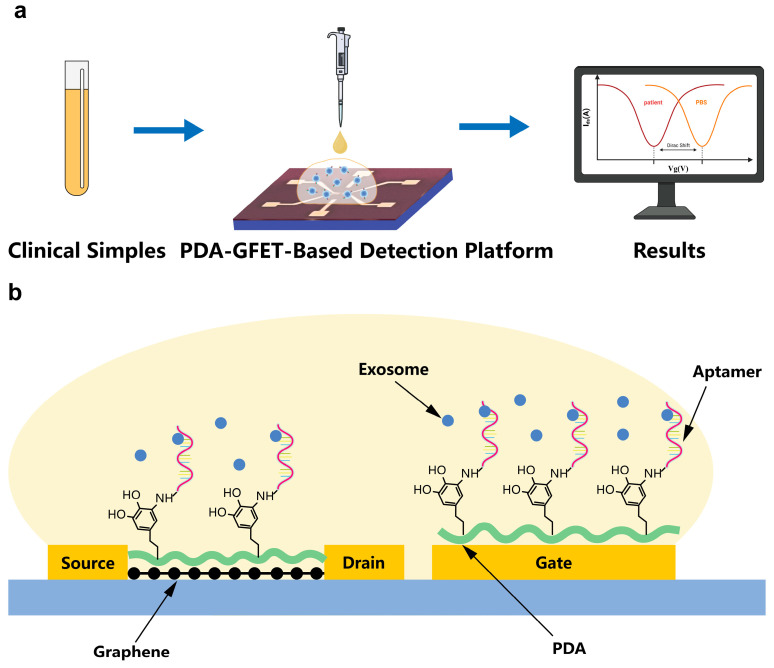
PDA-GFET-based detection platform. (**a**) The overall process of the PDA-GFET-based detection platform in clinical sample testing. (**b**) The planar-gate GFET functionalized for exosome detection using PDA and aptamer.

In this study, we developed a portable sensing platform based on polydopamine self-assembled film-functionalized graphene field-effect transistor (PDA-GFET) for rapid detection of colon cancer-derived exosomes in clinical samples, enabling detection within 10 min (Figure 1a). The platform consists of a planar-gate GFET coupled with a portable measurement system. For the overexpression of EpCAM, the membrane protein of colon cancer-derived exosomes, we chose EpCAM aptamer for specific capture to achieve rapid detection of colon cancer-derived exosomes. Moreover, polydopamine was used to cover the surfaces of the source–drain graphene channel and the gold gate electrode surface, forming a self-assembled film (Figure 1b). This not only inhibits the non-specific adsorption on the graphene surface but also functionalizes the gate, thereby enhancing the sensing area and realizing dual-area detection on both the source–drain channel and the gate electrode. The experimental results show that the developed PDA-GFET-based detection platform can detect EpCAM-targeted exosomes with high sensitivity, with a detection limit as low as 112 particles/mL, and successfully achieved statistical differentiation between healthy controls and colon cancer patients (*p* < 0.001). Thus, our sensing platform is expected to provide a new detection tool for the early and rapid diagnosis of colon cancer.

## 2. Materials and Methods

### 2.1. Materials

The dry oxygen-oxidized silicon wafers and chemical vapor deposition (CVD) monolayer graphene were purchased from Nanjing MKNANO Tech. Co., Ltd. (www.mukenano.com) (Naning, China). Dopamine hydrochloride, Tris-HCl solution, acetone, anhydrous ethanol, and ethanolamine were obtained from Sigma-Aldrich. PBS and fetal bovine serum were sourced from Macklin. The SW480 colorectal cancer cells were obtained from BNCC (BeNa Culture Collection) (Xinyang, China). The aptamer for EpCAM detection (5′-NH_2_-CAC TAC AGA GGT TGC GTC TGT CCC ACG TTG TCA TGG GGG GTT GGC CTG-3′) was synthesized and purified by Sangon Biotech Co., Ltd. (Shanghai, China).

### 2.2. Design and Fabrication of GFET Biosensor

The fabrication process of the GFET biosensor is consistent with the nanofabrication techniques used in our previous studies [41,42,43]. Briefly, Ti/Au thin films (5 nm/30 nm) were deposited and patterned on a silicon wafer by electron-beam evaporation to define the source, drain, and gate electrodes. In order to remove possible organic contaminants from the surface, the sensors were then treated in a plasma cleaner. The monolayer graphene with a PMMA protective layer was transferred to the source–drain electrode using a solution transfer method, where the graphene was precisely aligned as a conductive channel. After the transfer, the GFET device was soaked in acetone for 4 h to dissolve the PMMA protective layer, exposing the graphene surface. Throughout the preparation process, the electrode surfaces were cleaned with DI water and dried with nitrogen to ensure cleanliness and avoid contamination. These steps ensure the high quality and precise functionalization of the GFET sensor.

### 2.3. Preparation and Functionalization of PDA Self-Assembled Film

Tris-HCl buffer solution at a concentration of 1 M and pH 8.5 was added to DI water to prepare a Tris-HCl solution at a concentration of 10 mM. One mg of dopamine hydrochloride was weighed and dissolved in 10 mL of Tris-HCl solution at a concentration of 10 mM to allow dopamine to undergo a self-polymerization reaction to form polydopamine (PDA), and stirred with sufficient shaking. Then, 80 μL of the mixed solution of dopamine hydrochloride and Tris-HCl was withdrawn and added dropwise to the PDMS reaction vessel on the surface of the GFET, ensuring that the mixture fully covered the graphene between the source–drain and the gate. It was left at room temperature for 10 h to complete the formation of the polydopamine self-assembled film. Next, the sensor surface was rinsed using DI water and blown dry with nitrogen, and the PDA self-assembled film preparation was completed.

Subsequently, the device was immersed in a 1 μM EpCAM aptamer solution and incubated for 10 h. The NH_2_ at the 5′ end of the aptamer reacted with the PDA through a Schiff base reaction and Michael addition [44,45], thereby covalently attaching the aptamer to the device surface. Finally, the devices were immersed in a 100 mM ethanolamine solution for 1 h to quench other unreacted functional groups on the polydopamine film. Afterwards, the devices were rinsed using DI water and dried with nitrogen. Finally, the prepared sensors were stored at 4 °C for backup.

### 2.4. Colon Cancer-Derived Exosome Purification

First, the supernatant of cultured SW480 cells was collected and centrifuged at 400× *g* for 10 min to remove cellular debris and large particulate impurities. Next, the supernatant was transferred to a new centrifuge tube and centrifuged at 3000× *g* for 15 min to remove cell debris. Subsequently, the supernatant was filtered using a 0.45 μM membrane to remove microscopic impurities. To precipitate exosomes, initial enrichment was performed by centrifugation at 10,000× *g* for 40 min. To improve the purity of exosomes, the precipitate was resuspended in PBS buffer and centrifuged at 120,000× *g* for 90 min to further purify the exosomes. Finally, the purified exosome precipitate was resuspended in 500 μL of PBS and stored at −80 °C for backup.

## 3. Results

### 3.1. Characterization of PDA-GFET and Exosomes

**Figure 2:**
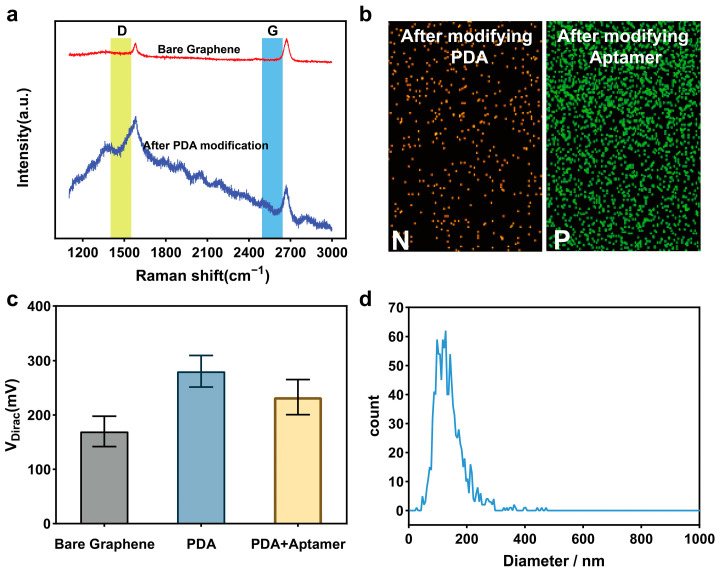
Characterization of PDA-GFET and exosomes. (**a**) Raman spectra of the bare graphene and after PDA modification. (**b**) EDS shows successful functionalization of PDA and aptamer. (**c**) Dirac curve response signal in functionalization processes. (**d**) Nanoparticle tracking analysis of purified exosomes.

The modification of the graphene surface using PDA was confirmed by Raman spectroscopy (Figure 2a). In bare graphene, the D peak is almost undetectable, indicating a low defect content. However, after PDA modification, the D peak was significantly enhanced, reflecting the fact that the modification of PDA introduced defects or heteroatom structures and coupled them to the graphene surface. This change indicates that the PDA modification alters the structural characteristics of graphene. Meanwhile, the shape and intensity of the G peak also change after PDA modification, further indicating that the PDA modification affects the electronic structure of graphene. Using an energy dispersive spectrometer (EDS) to analyze the elements on the graphene surface, we can clearly find that there is a significant increase in nitrogen after modification of PDA and phosphorus after modification of aptamer, which provides direct evidence of the successful modification of PDA and aptamer on the graphene surface (Figure 2b).

To further validate the functionalization of the graphene surface, we measured the transfer characteristics of the graphene (Figure 2c). The shift of the Dirac point (V_Dirac_) was selected as a characterization indicator. The results show that after the modification of the PDA self-assembled film, V_Dirac_ significantly shifted to the right, from 170 to 180 mV. This suggests that the modification of PDA introduces p-type doping, which alters the electronic properties of graphene. Furthermore, after aptamer modification, the Dirac point shifted left to around 230 mV, indicating that the aptamer modification induces n-type doping of graphene. These results fully demonstrate the successful functionalization of the biosensor through PDA self-assembled films and aptamers.

Exosome characterization was performed using nanoparticle tracking analysis (NTA) on exosomes extracted from the SW480 cell line via ultracentrifugation, analyzing the relationship between particle size and relative concentration (Figure 2d). The results showed that the concentration of purified exosomes was 5.3 × 10^10^ particles/mL, and the particle size distribution was mainly concentrated between 80 and 150 nm, which is consistent with the results reported in the literature [46,47,48]. These experimental results indicate that the purification of exosomes was successful.

### 3.2. Detection of Colon Cancer-Derived Exosomes in PBS

**Figure 3:**
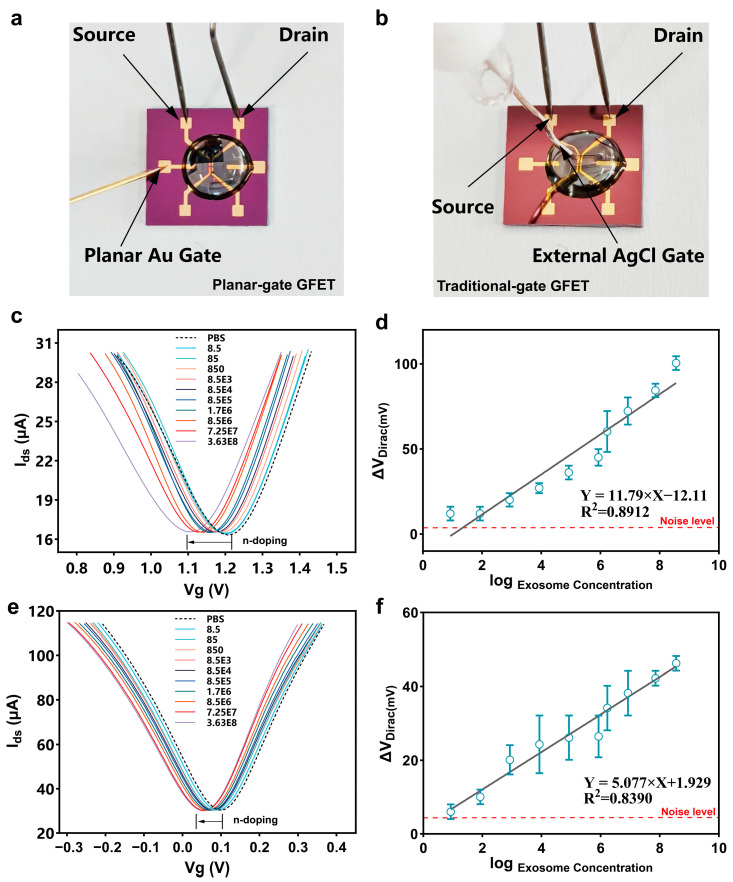
Detection of colon cancer-derived exosomes in PBS. Photos of the planar-gate GFET with the Au planar gate (**a**) and a traditional-gate GFET with an external AgCl gate (**b**). The Dirac characteristic curve (**c**) and linear fitting (**d**) of the planar-gate PDA-GFET for exosome detection in PBS. The dashed line represents the average change of the Dirac point after 10 times measurements of the device in 1× PBS; the Dirac characteristic curve (**e**) and linear fitting (**f**) of the planar-gate PDA-GFET for exosome detection in PBS. The dashed line represents the average change of the Dirac point after 10 times measurements of the device in 1× PBS.

To analyze the sensitivity and other performance characteristics of the PDA-GFET-based detection platform (Figure 3a), we diluted colon cancer-derived exosomes into different concentration gradients (from 8.5 to 3.63 × 10^8^) and applied them sequentially to the functionalized PDA-GFET-based detection platform. As the exosome concentration increased, the Dirac point shifted to the left (Figure 3c). This phenomenon is attributed to the aptamer binding with the negatively charged colon cancer-derived exosomes, forming a compact and stable G-quadruplex structure [49], which brought the exosomes close to the graphene surface and altered the electrical conductivity of graphene, resulting in an increase in the net carrier density of the graphene surface, which led to the creation of n-type doping and ultimately shifted the Dirac point to the left. Similarly, the aptamer pulls the negatively charged exosomes towards the gate surface, affecting the gate potential and enhancing the Dirac point shift signal. Under the combined effect of both, V_Dirac_ decreased by 104 mV from 1.206 V to 1.109 V. This response result is consistent with other results reported in the literature [50,51].

Figure 3d demonstrates the linear relationship between the log concentration of exosomes and ΔV_Dirac_(ΔV_Dirac_ = V_Dirac of PBS_ − V_Dirac of exosomes_), with a correlation coefficient R^2^ of 0.8912. The average noise level after 10 times measured is 4.02 mV. On the basis of the signal that exceeds the noise level by 3-fold (SNR = 3), the limit of detection (LoD) was estimated to be 112 particles/mL when detecting a signal more than 3 times the baseline. This indicates that the PDA-GFET detection platform has a high sensitivity and a low limit of detection, which is much lower compared to most of the existing exosome detection methods (Table 1).

To further verify whether the aptamer on the planar gate successfully captured exosomes and whether the sensor signal was enhanced compared to, we performed control experiments with the traditional-gate GFET (Figure 3b), and the Dirac transfer characteristic curves are shown in Figure 3e. As the exosome concentration increased from 8.5 to 3.63 × 10^8^, the V_Dirac_ decreased from 0.09 V to 0.053 V, a decrease of 44.22 mV, indicating that its signal response was significantly smaller than that of the planar-gate GFET. The correlation coefficient R^2^ was 0.8390. The average noise level after 10 times measured is 5.36 mV. On the basis of the signal that exceeds the noise level by 3-fold (SNR = 3), LoD was calculated to be 612 particles/mL (Figure 3f).

### 3.3. Detection of Colon Cancer-Derived Exosomes in Undiluted Serum

**Figure 4:**
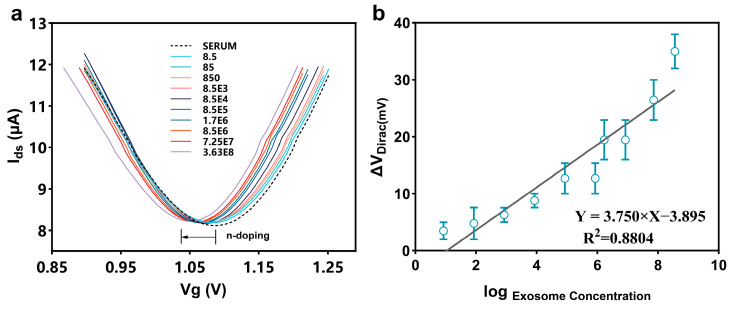
The PDA-GFET-based detection platform detects exosomes at different concentrations in undiluted FBS with its Dirac characteristic curve (**a**) and linear fitting (**b**).

To further validate the anti-interference ability of the PDA-GFET-based detection platform in real physiological environments, we performed experiments in undiluted fetal bovine serum (FBS). By diluting colon cancer-derived exosomes into different concentration gradients and adding them dropwise to the GFET surface, the measurement results are shown in Figure 4a. The FBS control sample induced a response of 3.79 mV. Different concentrations of exosomes resulted in a significant change in Dirac voltage of 38 mV, and the trend of the signal change was highly consistent with the results of the tests in PBS. The regression equation is Δ*V*_Dirac_ = 3.995 log C − 5.474 with a correlation coefficient R² of 0.8514, which is only 4.4% different from the test results in PBS, as shown in Figure 4b. This indicates that the performance of the PDA-GFET-based detection platform in FBS is in high agreement with the values tested in PBS.

The modification of the polydopamine self-assembled film effectively reduced the non-specific molecular interactions with the sensor surface, successfully suppressing non-specific adsorption. On the other hand, the polydopamine film was directly applied to the aptamer’s branching, which functionalized the gate and further enhanced the response signal. As a result, the PDA-GFET-based detection platform was able to achieve exosome detection in undiluted physiological media, demonstrated good anti-interference ability, and provided a new approach for exosome detection in real biological samples.

### 3.4. Integrated Portable Platform for Clinical Sample Detection

**Figure 5:**
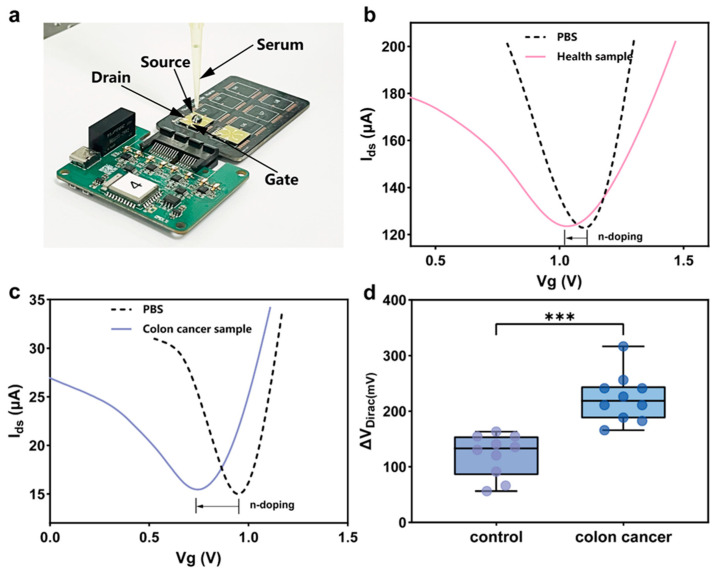
PDA-GFET-based detection platform for clinical sample detection in colon cancer patients. (**a**) Field test photo of the PDA-GFET-based detection platform; Dirac response curves of healthy samples (**b**) and colon cancer samples (**c**); (**d**) Analysis of the significant difference in response signals between healthy samples and colon cancer samples (*** represents *p* < 0.001).

Next, we used the PDA-GFET-based detection platform to detect colon cancer-derived exosomes in real clinical samples. The test photograph is shown in Figure 5a. Serum samples were collected from 10 healthy individuals as the control group and 10 colon cancer patients as the experimental group. To ensure the accuracy and reproducibility of the experiments, equal volumes of serum from each clinical sample were applied to the PDA-GFET-based detection platform to measure their response signal curves, as shown in Figure 5b,c.

During the measurement process, to ensure baseline stability of the PDA-GFET-based detection platform, we repeated signal measurements for a PBS solution and ensured that its Δ*V*_Dirac_ value remained within 5 mV. After several experiments, we verified that the signal change of the PBS solution was very weak and almost negligible, so we used this as a baseline to further observe the response signal change of each clinical sample.

For the 20 clinical samples tested, we plotted their response signal curves as shown in Figure 5d. The results show that the response signals of serum samples from colon cancer patients on the PDA-GFET-based detection platform are significantly higher than those of healthy controls. This difference was statistically significant (*p* < 0.001), further validating that the PDA-GFET-based detection platform can effectively distinguish the exosome levels of colon cancer patients and healthy individuals. Specifically, during tumor progression, colorectal cancer cells release exosomes through various mechanisms, leading to a markedly higher number of colon cancer-derived exosomes in the serum samples from cancer patients compared to the healthy population.

## 4. Discussion

The above experimental results demonstrate that the planar-gate PDA-GFET-based detection platform exhibits higher sensitivity and lower detection limits compared to the traditional-gate GFET. Although both types are solution-gate GFETs, due to the instability of the gate position, the traditional-gate GFET is susceptible to external interference, resulting in unstable measurement signals, and this instability significantly affects the accuracy and repeatability of the detection results. In addition, the traditional-gate GFET can only achieve functionalization of the graphene channel due to structural defects, ignoring the equally important role of gate functionalization in enhancing sensing efficiency. In contrast, the planar-gate PDA-GFET eliminates the instability of the external gate structure by directly integrating the gate into the source–drain electrode plane, significantly improving the device’s electrical stability. Additionally, the PDA self-assembled thin film can directly and simultaneously functionalize both the gate and the graphene channel [43,44], enabling both the functionalized gate and graphene channel to specifically bind with target biomolecules on the surface, thereby increasing the sensing area and further enhancing the response signal. These improvements enable the planar-gate PDA-GFET to exhibit superior performance in terms of sensitivity, detection limit, and stability and are particularly suitable for efficient detection of target molecules at low concentrations. At the same time, Figure 5d demonstrates that the PDA-GFET-based detection platform is highly sensitive and selective and can effectively differentiate exosome levels in serum samples and accurately identify differences between colon cancer patients and healthy controls. This makes the PDA-GFET-based detection platform a promising detection tool for the early diagnosis of colon cancer.

## 5. Conclusions

In conclusion, this study presents a planar-gate graphene field-effect transistor-based detection platform based on polydopamine functionalization to achieve rapid, label-free detection of colon cancer-derived exosomes in serum. With remarkable sensitivity and specificity, the platform enables colon cancer detection by expression of the surface-specific membrane protein EpCAM within 10 min. Compared to conventional methods, the PDA-GFET-based detection platform not only reduces the detection limit but also improves signal stability by eliminating gate position instability. The modification of polydopamine self-assembled film on the surface of graphene and gate effectively suppressed the non-specific adsorption, which further improved the detection performance. By detecting exosomes in clinical samples from healthy individuals and colon cancer patients, the experimental results showed significant differences in signals between the two, demonstrating the potential of this platform for clinical applications. This PDA-GFET-based detection platform provides an innovative, rapid, and low-cost assay for the early diagnosis of colon cancer and is expected to be an important tool for future clinical screening and personalized treatment.

## Figures and Tables

**Table 1:** Comparison of the PDA-GFET-based detection platform with existing methods.

Sensing Method	Probe	Test Time	LOD	Reference
Electrochemical biosensor	CD9 antibody	N/A	2 × 10^5^ exosomes/mL	[52]
Electrochemical biosensor	CD63 aptamer	80 min	5 × 10^3^ exosomes/mL	[53]
colorimetric aptasensor	CD63 aptamer	40 min	5.2 × 10^5^ exosomes/mL	[54]
colorimetric aptasensor	CD63 aptamer	10 min	7.7 × 10^3^ exosomes/mL	[55]
FET biosensor	CD63 antibody	30 min	2 × 10^4^ exosomes/mL	[56]
GFET biosensor	EpCAM aptamer	10 min	112 exosomes/mL	This work

## Data Availability

The data are contained within the article.

## Abbreviations

The following abbreviations are used in this manuscript:

PDA	Polydopamine
GFET	Graphene-based Field-Effect Transistor
EpCAM	Epithelial Cell Adhesion Molecule
PMMA	Polymethyl Methacrylate
NTA	Nanoparticle tracking analysis
Tris-HCl	Tris(hydroxymethyl)aminomethane hydrochloride
